# Liver Transplantation Update: 2014

**DOI:** 10.5005/jp-journals-10018-1144

**Published:** 2016-07-09

**Authors:** Serkan Dogan, Ahmet Gurakar

**Affiliations:** 1 Department of Gastroenterology, Johns Hopkins School of Medicine, Maryland, United States; 2 Division of Gastroenterology and Hepatology, Johns Hopkins School of Medicine, Maryland, United States

**Keywords:** Acute liver failure, Cardiac calcium score, Cirrhosis, Coronary artery disease, Hepatitis C, Hepatocellular carcinoma, Liver transplantation, Milan criteria, Model for end-stage liver disease, UCSF criteria.

## Abstract

The first liver transplantation (LT) was performed by Thomas E Starzl five decades ago, and yet it remains the only therapeutic option offering gold standard treatment for end-stage liver disease (ESLD) and acute liver failure (ALF) and certain early-stage liver tumors. Post-liver transplantation survival has also dramatically improved over the last few decades despite increasing donor and recipient age and more frequent use of marginal organs to overcome the organ shortage. Currently, the overall 1 year survival following LT in the United States is reported as 85 to 90%, while the 10 years survival rate is ~50% (http://www.unos.org). The improvements are mainly due to progress in surgical techniques, postoperative intensive care, and the advent of new immunosuppressive agents. There are a number of factors that influence the outcomes prior to transplantation. Since 2002, the model for end-stage liver disease (MELD) score has been considered a predicting variable. It has been used to prioritize patients on the transplant waiting list and is currently the standard method used to assess severity in all etiologies of cirrhosis. Hepatocellular carcinoma (HCC) is the most common standard MELD exception because the MELD does not necessarily reflect the medical urgency of patients with HCC. The criteria for candidates with HCC for receiving LT have evolved over the past decade. Now, patients with HCC who do not meet the traditional Milan (MC) or UCSF criteria for LT often undergo downstaging therapy I an effort to shrink the tumor size. The shortage of donor organs is a universal problem. In some countries, the development of a deceased organ donation program has been prevented due to socioeconomic, cultural, legal and other factors. Due to the shortage of cadaveric donors, several innovative techniques have been developed to expand the organ donor pool, such as split liver grafts, marginal- or extended-criteria donors, live donor liver transplantation (LDLT), and the use of organs donated after cardiac death. Herein, we briefly summarize recent advances in knowledge related to LT. We also report common causes of death after liver transplant, including the recurrence of hepatitis C virus (HCV) and its management, and coronary artery disease (CAD), including the role of the cardiac calcium score in identifying occult CAD.

**How to cite this article:**

Dogan S, Gurakar A. Liver Transplantation Update: 2014. Euroasian J Hepato-Gastroenterol 2015;5(2):98-106.

## PRIORITIZATION OF ORGANS: MODEL FOR END-STAGE LIVER DISEASE AND EXCEPTION POINTS

Prior to February 27, 2002, organ allocation for LT was prioritized based on the child-turcotte-pugh (CTP) score. This scoring system used subjective parameters, such as ascites and encephalopathy, to estimate the short-term risk of death in patients who were on the transplant list. It was subjected to manipulation, leading to the inappropriate usage of organs. The system was also depended ‘on the list’ waiting times for organ allocation.^3^ Because of these problems, a more justifiable donor organ allocation system became necessary, and ultimately the MELD score replaced the CTP. The MELD score makes use of objective parameters to anticipate the short-term risk of death in patients who are on the transplant list.^1^ It was originally validated with the aim of predicting survival after transjugular intrahepatic portosystemic shunt (TIPS), but now, it is also used to objectively quantify the severity of recipient disease and to prioritize organ allocation in patients awaiting deceased-donor liver transplantation (DDLT).^2,4^ Model for end-stage liver disease has significantly reduced mortality on the waiting list. Since the application of MELD by The United Network for Organ Sharing (UNOS) on February 27, 2002, the rate of waiting list mortality significantly dropped from 30% in 2001 to 15% in 2005.^5^ This mathematical prioritization model uses the total serum bilirubin concentration, the International Normalized Ratio (INR) for the prothrombin time, and the serum creatinine concentration to determine a score from 6 to 40, and predicts 3 months mortality risk.^6^

MELD = 9.57 log [Creatinine (mg/dL)] + 3.78 log [Bilirubin (mg/dl)] + 11.20 ln [International Normalized Ratio] + 6.43.

Patients with the highest MELD scores receive liver transplants regardless of how long they have been on the transplant waiting list. However, there are some special conditions that correlate with survival and stage liver disease that may result in impaired survival, but are not directly accounted for in the MELD scoring system. Some of these conditions, such as HCC, hepatopulmonary syndrome, primary hyperoxaluria, familial amyloid polyneuropathy, cystic fibrosis with progressive pulmonary compromise, portopulmonary syndrome, and cholan-giocarcinoma (after receiving an approved chemoradia-tion protocol) have been identified and termed ‘MELD exception points’.

Other considerations, such as serum sodium concentration (especially in those with low MELD scores), donor age (D-MELD), and frequent cholangitis in those with primary sclerosing cholangitis have been discussed to be important factors that predict prognosis,^7-9^ but they are still not considered standard MELD exception points by international consensus.

## UPDATES IN CARDIAC CLEARANCE PRIOR TO LIVER TRANSPLANTATION

Pretransplant screening and management is important for all patients on the waiting list. The evaluation process should cover underlying risk factors that predispose recipients to cardiovascular disease. Multiple studies have shown associations between CAD and ESLD, and some have reported an increased incidence of up to 27%.^10^ Those with ESLD and angiographically verified CAD (pretransplant) have increased mortality after LT.^11^ Therefore, preoperative cardiac screening prior to LT may reduce adverse events with associated CAD. Myocardial perfusion imaging with single-photon emission tomography and dobutamine stress echocardiography is often used in the evaluation of CAD. The coronary calcium score (CCS) is also used to estimate the risk of cardiovascular events in persons without previous CAD. Taydas et al conducted a study to determine the utilization of CCS to predict the presence of CAD in asymptomatic candidates with a history of major cardiac risk factors. They found that a CCS above 250 can indicate that a patient may have early coronary disease, and these patients may benefit from further investigation with a cardiac catheterization.^12^

In addition, coronary-artery calcium levels can be used for cardiovascular risk prediction in high risk patients with ESLD. The procedure may reduce unnecessary cardiac catheterizations.

### Hepatocellular Carcinoma

Hepatocellular carcinoma is a major global health problem. It is the fifth most common form of cancer in men and the seventh in women.^13^ Hepatocellular carcinoma is a primary malignancy of hepatic origin, and its epidemiology varies in different parts of the world. While HCV and alcoholic liver disease causes most of the HCC in Western countries, hepatitis B virus (HBV) infection is the leading cause in Southeast Asia and sub-Saharan Africa, where it is endemic.^14^ Other possible HCC risk factors include hereditary hemochromatosis, alpha1-antitrypsin deficiency, autoimmune hepatitis, certain porphyrias and Wilson’s disease. These risk factors can lead to the formation and progression of cirrhosis, which is present in 80 to 90% of HCC patients. However, HBV and HCV have oncogenic potential, and they can play a significant role in the risk of HCC, even in the absence of cirrhosis.^15^ An HCC diagnosis is typically given during routine screenings in risky populations who are infected with hepatitis virus and have cirrhosis, although in some cases, the diagnosis is made only after the patient develops a symptomatic lesion.

Because their clinical cases are often very complex, treatment of patients with HCC should be managed by a multidisciplinary team that includes a hepatologist, hepatobiliary surgeon, transplant surgeon, radiologist, pathologist and oncologist. Hepatocellular carcinoma has many potentially curative therapeutic approaches, including liver resection, transplantation and local ablation. The choice of treatment should be determined based on the stage of HCC, the severity of the underlying liver disease, the availability of treatment resources, and clinical expertise. The success of liver transplantation (LT) in HCC is based on the tumor stage, and patients with widespread disease often have very poor outcomes, while most patients with small tumors are often fully cured. Tumors are evaluated with the MC and Barcelona Clinic Liver Cancer (BCLC) staging system.^16,17^ The BCLC staging system is the standard means of assessing the prognosis for patients with HCC and incorporates the patient’s performance status, cancer symptoms, and liver function as determined by the Child Pugh Turcotte classification system and the TNM stage.

Surgical resection is recommended as the first-line therapeutic option for individuals without cirrhosis, which is defined by the presence of asymptomatic solitary HCC with a diameter of <2 cm and no vascular or distant metastases.^18^ Currently, the 5-year overall survival and recurrence rates after liver resection of cases with very early-stage HCC are 70 and 68%, respectively.^19^ Some authors have suggested that radiofrequency ablation (RFA) is a better option than surgical resection in terms of overall survival and because it has fewer side effects.^20,21^ After hepatic resection of patients with cirrhosis, the cumulative 5-year risk of recurrence can reach as high as 70% because the underlying chronic liver disease that caused the premalignant condition is still present. Liver transplantation is also the first-line therapeutic option for patients with decompensated (Child-Pugh B or C) cirrhosis because it increases long-term survival in selected candidates. The American Association for the Study of Liver Diseases (AASLD) and the European Association for the Study of the Liver (EASL) have proposed that MC should be used to determine whether patients with a solitary nodule with a diameter no greater than 5 cm, or no more than three nodules, of which the largest is less than 3 cm without extra hepatic involvement (including absence of microvascular invasion) are eligible for LT.^22^

Risky patients with HCC are screened based on ultrasonography and the measurement of serum alpha-fetoprotein (s-AFP) levels. The sensitivity and specificity of the s-AFP measurement increase substantially in patients with advanced liver fibrosis when blood s-AFP levels are above 400 ng/ml.^23^ The methods most commonly used to establish a diagnosis in cirrhotic patients suspected of having HCC and for preoperative staging are dynamic CT or dynamic MRI with the presence of arterial enhancement followed by washout on portal venous or delayed imaging.^24^

Liver transplantation should be reserved for HCC patients who have a predicted 5-year survival that is comparable to that of non-HCC patients. This rule should be in place so that HCC patients do not have an advantage over non-HCC patients awaiting transplantation.^26^ According to the MC, the 5-year survival rate is now approximately 70%, in contrast to the rate of approximately 50% when LT was performed using unrestricted indications.^25^ In a recent systemic review of 90 studies (Mazzaferro et al) involving 17780 post-transplant HCC patients observed over a 15-year period, it was determined that the MC are an independent prognostic factor for outcome after LT.^27^ In the United States, the MC were adopted by the UNOS to guide patient selection for cadaveric orthotopic liver transplantation (OLT) for cases with HCC. Patients who meet the MC are given an extra 22 MELD points. They also receive an additional three points for every subsequent 3 months spent on the waiting list. The purpose of adding the extra points is to shorten their waiting time and to avoid tumor progression and the risk of waitlist dropout.

Due to the increasing shortage of donor livers and waitlist dropout in liver transplant candidates with HCC within MC, transplant centers are in need of new criteria. Among the many candidates, only the University of California San Francisco (UCSF) criteria (solitary tumor ≤6.5 cm, three nodules at most with the largest being ≤4.5 cm, and cumulative tumor diameter ≤8 cm) have been prospectively validated by the proposer group.^27,28^ Currently, the UNOS has not adopted the UCSF criteria due to limited organ availability.

Due to the shortage of donor organs, liver transplant candidates often have a long wait time before LT. Increased wait-list time may lead to dropout. The so-called ‘bridging therapy’ is used in HCC patients to reduce the wait-list dropout rate before transplantation, to decrease recurrence of HCC after transplantation, and to improve the post-transplant overall survival.^29^ Among the many treatment options, locoregional therapies, such as radio-frequency ablation (RFA) and transarterial chemoembo-lization (TACE) are the most commonly used bridging treatment choices prior to transplantation. In theory, surgical resection should be used as a first line bridging therapy to LT because it allows for the best pathological analysis and tumor control. However, most transplant centers prefer locoregional therapies, because they have less periprocedural risk, fewer costs, and less postoperative complications.^30^ Liver resection can be considered for patients with single exophytic or subcapsular neoplasms and preserved liver function. The optimal strategy for MC in patients awaiting LT should be transplant within 6 months without pre-transplant therapy.^31^ If a longer time period is required, AASLD and EASL suggest that the bridging treatments should be used to prevent tumor progression.^32^

When a patient exceeds the MC or UCSF criteria, some centers use downstaging therapy with alcohol injection, TACE, RFA, transarterial radioembolization (TARE), or liver resection. The aim of the therapy, in addition to reducing the tumor size and number, is to improve the patient’s suitability for transplantation.^33^ Two prospective studies reported that survival following LT in patients with large tumors that were successfully downstaged within the MC or UCSF criteria is similar to that of patients who initially met the criteria for transplantation.^34,35^

### Liver Transplantation using Marginal- or Extended-Criteria Donors, Organs Donated after Cardiac Death, Split Liver Grafts, and Live Donor Liver Transplantation

The requirements for organ transplantation have rapidly increased worldwide over the past two decades, and the number of patients on the transplant waiting list increase continually. Innovative approaches have been developed to close the gap between supply and demand, and include the use of split liver grafts, domino transplantation, an increased use of donors after circulatory death (DCD), higher risk donors after brain death (DBD), grafts and live donor liver transplants.

### Extended-Criteria and Marginal Donors

Over the last few decades, LT has become a routinely applicable therapy for a widening group of patients with end-stage liver disease (ESLD). However, while the demand has increased exponentially, the supply (number of procedures performed) has increased only modestly. Therefore, extended criteria donors are sometimes used for LT, and these include steatotic livers, older donors, donors with positive serology, split livers, and DCD. It is known that liver transplant patients with HCV often experience HCV recurrences in the graft. Approximately 50% of transplanted livers will develop cirrhosis at 10 years.^36^ Therefore, transplants from the HCV+ donor pool to HCV+ recipients are now routinely performed. Results from some previous studies suggest that there is no statistical difference between recipients with HCV using HCV positive or HCV negative allografts.^37^ A donor graft biopsy prior to the procedure is not recommended, except in cases where the donation was made after cardiac death, or in cases where multiple extended criteria donor factors are involved.^38^

Transplantation of grafts from HBsAg-/HBcAb+ donors can be safely used to expand the donor pool, and their use in HBV-naive recipients does not increase mortality or graft loss. However, prophylactic antiviral therapy (hepatitis B immunoglobulin, alone or in combination with lamivudine) should be used in these cases to prevent the potential development of *de novo* HBV caused by latent infection that can be reactivated in the setting of immunosuppression, especially in HBsAg-/ HBcAb- patients, almost all of whom have a recurrence without prophylaxis.^39,40^

The scarcity of organ donors has forced many liver transplant centers to expand their criteria, such as including older age donors. However, there is no consensus on the age limit for their acceptance. Recently, a single-center study of 3,751 adult recipients with irreversible liver failure indicated that among the seven donor variables (age, graft type, cardiac arrest, sex, hospital stay, serum sodium, and number of vasopressors), only donor age affected post-transplant survival, with the highest risk in donors older than 60 years.^41^ Donor age is also a strong predictor for graft loss in liver transplant patients with HCV and those who are over the age of 60.^42^

### Donation after Circulatory Death

Donor quality is one of the most important causal factors in peritransplantation and post-transplantation organ function. Of utmost importance is the use of standard criteria donors (i.e. good quality donors). Deceased heart-beating donor (DBD) grafts were used in most of the liver transplants in the 1990s. However, the scarcity of organ donors has forced many liver transplant centers to expand their criteria for the acceptance of marginal grafts, as well as to perform DCD. Now, DCD also includes non-heart-beating donors (NHBD) or death after cardiac death donors, which have become important methods of organ donation during the last decade.^43^

Donar after circulatory death donation occurs after the declaration of death and is based on cardiorespira-tory criteria. This is in comparison with donation after DBD, in which neurological criteria are used. According to Maastricht criteria, four types of DCD donors have been defined.^44^ Dead on arrival and unsuccessful resuscitation (Categories I and II, respectively) comprise the ‘uncontrolled donors’. Awaiting cardiac arrest and cardiac arrest in brain-dead donor (Categories III and IV, respectively) comprise the ‘controlled donors’. Controlled donors are suitable (probably Maastricht category III DCD donors) because they have a short and predictable ischemia time, and, with respect to uncontrolled donors, a better chance of recovery. Ischemia time can be divided into cold ischemia time (CIT) (from cross-clamp to the start of perfusion) and warm ischemia time (WIT) (from cessation of cardiopulmonary support to perfused with cold crystalloid solution). Donar after circulatory death livers are more susceptible to damage than DBD livers, which is most likely due to hypoperfusion that occurs during the agonal phase (from hemodynamic changes to asystole). Both times must be kept as short as possible to reduce the chance of a negative outcome. Warm ischemia time and CIT are well defined as 20 to 30 minutes and 8 to 10 hours, respectively.^45^

In brain death, donor warm ischemia is eliminated because pulsatile perfusion has remained largely unchanged during retrieval. Thus, effective natural organ perfusion protects grafts from the deleterious effects of warm ischemia and is critical to the future of recipient mortality and morbidity. Several data suggest that DCD organs have higher rates of primary nonfunction, vascular complications, and ischemic cholangiopathy and retransplantation when compared with DBD. A meta-analysis of studies from 1950 to 2009 showed that those in the DCD group were more likely to have biliary complications (2.4 times greater risk), and were more likely to have ischemic cholangiopathy (10.1 times greater risk than the DBD group). In addition, the rate of 1-year graft failure, 1-year patient mortality, primary nonfunction, 3-year graft failure and retransplantation was increased among patients who had been DCD.^46^

In addition, despite preservation of DCD livers by hypothermic machine perfusion (MP), which had been shown to have advantageous safety features in many experimental studies compared with static cold storage (SCS), only SCS is clinically approved, and MP is still being tested in experimental preclinical research.^47-49^ Pharmaceutical interventions include vasodilators, fibri-nolytic agents, antioxidants, antibiotics, and hormones during SCS. These interventions have received considerable attention to date for the prevention of vasospasm, thrombus formation in the microcirculation, and the risk of colonic bacterial contamination secondary to transloca-tion of organisms during the warm ischemic period.^50^

Thus, satisfactory results can be achieved in DCD donors that provide donor recipient compatibility, minimization of warm-cold ischemia, and good postoperative care.

### Living Donor Liver Transplantation

Many organ transplant centers suffer from a shortage of suitable donors. Inconsistencies between the number of patients awaiting LT and the number of provided donor liver transplants have led to the expansion of donor criteria. These expanded criteria include deceased donor grafts, split liver transplants, organs harvested after cardiac death, partial auxiliary liver transplant grafts, and living donation grafts. Living donor liver transplantation (LDLT) was first performed by Broelsch CE et al in 1989 in a 21-month-old baby who suffered from biliary atresia; this LDLT was performed for logistic reasons.^51^ Pediatric cadaveric organs were not as readily available as those from adult donors, and this is still true today. Other methods intended to expand the donor pool, such as the use of splitting or reducing a deceased adult donor organ, have become accepted practices.^52^ Prior to the use of LDLT, pediatric recipients had longer waiting-list times that were associated with a high risk of morbidity and mortality. Once the safety and efficacy of this therapy was determined in pediatrics, the practice of LDLT was extended to adults. The enthusiasm for LDLT increased exponentially from the late 1990s until 2001.^53^ In 2002, the number of living liver donors dropped significantly due to the first widely publicized death of a living donor.^54^ While the debate remains regarding the safety and appropriateness of adult-to-adult LDLT in the United States, some Asian countries (especially Turkey and Egypt) have experienced a continued rise in adult LDLT due to social norms and logistic difficulties that block cadaveric organ donation.^55,56^

The advantages of LDLT include a minimum waiting time, ability to determine immunologic similarities between the donors and recipients, and the ability to use a graft from a healthy donor with minimal ischemic time. Adem et al (from the European Liver Transplant Registry) reported that LDLT survival was comparable to that of after brain death full liver grafts at 1, 3 and 5 years.^57^ In addition, the LDLT can be performed during a period for low postoperative hepatitis C recurrence in patients with chronic HCV that have sustained virologic response.^58^

The risks of LDLT to the donor include infection, pleural effusion, bile leak, neuropraxia, reexploration, prolonged ileus, hernia, psychological complications, bowel obstruction and death. Results from the Adult-to-Adult Living Donor Liver Transplantation (A2ALL) Cohort Study indicated that recipients of LDLT had higher rates of complications (i.e. biliary leak, unplanned re-exploration and portal vein thrombosis) than did recipients of whole-organ deceased donor allografts.^59^ Studies of various medical groups also reported that donors with BMI ≥30, macrovesicular steatosis, increased age, prolonged operative time, and especially, intraoperative blood transfusion were at increased risk for complications.^59,60^

Most transplant centers use the right lobe of the liver in adult LDLT to provide adequate graft volume to fulfill all of the metabolic demands. However, the use of the right lobe may lead to impaired graft outflow because it interrupts the venous drainage of the middle hepatic vein (MHV). Postoperative congestion may result in early postoperative graft failure and recipient death. Several procedural modifications have been proposed to preserve MHV outflow drainage in a right lobe. These modifications include the use of modified or cadaveric grafts, and eliminating the resection of the donor MHV.^61,62^

Despite its various advantages, the incidence of biliary complication in right lobe LDLT is greater than that of DCD and pediatric left lateral lobe LDLT. Different anatomies between the left and right hepatic biliary systems, differential blood supplies of the graft ducts, and differing techniques for biliary reconstruction may be predisposing factors in the pathophysiological mechanisms of biliary complications. Most biliary complications after LDLT can be successfully treated by nonsurgical options, which include endoscopic retrograde cholan-giopancreatography and percutaneous transhepatic radiologic procedures.^63^

Although the complication rates are significantly higher in right lobe LDLT then left, the majority of studies have shown that right lobe grafts had better survival than left lobe grafts.^64^ The difference in recipient outcome (between right and left lobe donation) cannot be explained by size alone. Venous outflow and portal inflow may affect the outcomes. Flow modulating techniques, such as left portal and left hepatic vein shunts, hemipor-tocaval shunt, splenic artery ligation or embolization can ensure a higher rate of good outcomes by preventing the development of small-for-size syndrome.^65^

Potential donors are subject to screening processes based on blood type, age, weight, height, liver size, cardiac tests, laboratories, imaging, psychosocial issues, relationship to the patient and donor reluctance. It is important to pay close attention to these factors to ensure that the donor is medically, psychologically, and surgically fit.^66^

Despite the ongoing and increasing shortage of cadaveric livers, LDLT offers an unlimited donor organ supply. Donor safety is paramount, is an absolute priority, and should never be compromised. Procedures should be performed by experienced surgeons, and donors and recipients should be carefully selected.

### Split Liver

Split liver transplantation (SLT), which is the sharing of a donor liver from a cadaveric adult between a pediatric recipient and an adult recipient, is an irresistible option for expanding the donor organ pool. However, increased morbidity and technical difficulties in comparison with full size LT have been associated with the reduced use of the procedure.^67^ On the other hand, clinical studies have shown that SLT remains an important clinical procedure for transplant centers. In 2013, results from a large, single-center study from Birmingham were published. These results showed an 82% 10-year survival rate for pediatric recipients and a 62% 10-year survival rate for adult recipients. Despite differences in the rate of graft loss and technical complications between SLT and full size LT, SLT outcomes have improved significantly, and now, the prognosis is almost equivalent to that of full size liver transplantation.^68^

## RECURRENT HEPATITIS C FOLLOWING LIVER TRANSPLANTATION

In most countries, chronic HCV is one of the main causes of liver transplant indication. Furthermore, reinfection of the graft with HCV is practically inevitable, and is a clinical problem that needs to be resolved as it causes reduced graft and patient survival. It is a definite drawback for transplant centers because of the lack of treatment options with established reliability. Many studies have shown that patients with sustained viral response (SVR) before LT achieved transplant outcomes comparable to those of other indications.^69^ Recurrence occurs if plasma HCV ribonucleic acid (RNA) remains detectable during transplantation, and approximately 30% of these patients experience accelerated progression to cirrhosis within 5 years of LT.^70^ A number of factors associated with more rapid HCV recurrence and complications have been identified, and include high HCV RNA viral load in both serum and liver at the time of LT, genotype 1, female gender, older donor age, steatosis of the graft, degree of human leukocyte antigen (HLA) matching or the IL28b genotype of the donor and the recipient, and *de novo* donor-specific antibodies.^68,71^

Prospective studies have shown that pre-emptive treatment approaches (e.g. the routine use of prophylactic antiviral therapy before the development of biochemical and histological evidence of recurrent HCV infection) were not superior to the treatment of recurrent chronic hepatitis due to a low rate of SVR, side effects, and a high rate of discontinuation.^72^ In addition, outcomes of treatment with pegylated interferon therapies (dual or triple) in HCV recurrence recipients may be suboptimal, in part because of intolerance of side effects and potential drug-drug interactions.^73^ Clinically, relevant drug interactions have occurred with the use of first generation protease inhibitors. Telaprevir and Boceprevir magnify the therapeutic effect of calcineurin inhibitors by the CYP3A4 system.

These factors threaten to alienate many liver transplant providers. In light of this information, studies have revealed the need for new approaches for HCV patients with extensive comorbidities, including cirrhosis. In other words, the therapy should be simpler, safer, and more effective.^74^ Currently, many combinations of protease inhibitors (including NS5A) and polymerase inhibitors (with or without ribavirin) are being evaluated for toler-ability and efficacy.^75^ We have also become a part of a prospective, multicenter study of combination therapy with use of Simeprevir and Sofosbuvir in post-transplant HCV recurrence. Premature data from the ongoing trial indicates that the combination therapy is generally safe, effective and well tolerated. Thus, in the near future, the next generations of direct-acting antiviral agents will change the management of hepatitis C infection in liver transplant recipients. Clinical use will allow a substantial percentage of patients to be treated in a more effective way with more favorable clinical outcomes.

In conclusion, in spite of its inauspicious start five decades ago, liver transplantation has become the best therapeutic option for selected patients with irreversible liver failure. Over the past five decades, there have been substantial developments in the operative procedure and in the prevention of intraoperative problems, evolving immunosuppressive regimens, and improvements in organ procurement, preservation, and liver-allocation concepts^76^ Long-term overall survival has been improving, clinicians are encouraged to perform more transplantation procedures.
